# Determining the Treatment Strategy for Refractory Ulcerative Colitis Using Prostaglandin E-major Urinary Metabolite (PGE-MUM) Measurement

**DOI:** 10.7759/cureus.64637

**Published:** 2024-07-16

**Authors:** Ryosuke Miyamoto, Hitoshi Honma, Yu Masuda, Yoshinori Ito, Akihisa Okumura

**Affiliations:** 1 Department of Pediatrics, Aichi Medical University, Nagakute, JPN

**Keywords:** mirikizumab, biological agents, prostaglandin e-major urinary metabolite, cytokine profile, ulcerative colitis

## Abstract

Prostaglandin E-major urinary metabolite (PGE-MUM) is a valuable biomarker reflecting the cytokine profile. We encountered a case of a 14-year-old boy with pan-colitis-type ulcerative colitis who was unresponsive to steroids and infliximab. The patient’s clinical symptoms gradually deteriorated and surgical treatment was strongly considered because anti-inflammatory therapy was unlikely to be effective. PGE-MUM levels were markedly elevated, indicating a T-helper 17 (Th17)-like cytokine profile. Because an antibody against interleukin 23 (IL-23) was presumed to be effective, the patient was treated with mirikizumab, after which he achieved remission. In the present case, measurement of PGE-MUM levels was useful in selecting anti-cytokine treatments for severe ulcerative colitis.

## Introduction

Ulcerative colitis (UC) is a chronic inflammatory disease characterized by repeated remissions and exacerbations. Cases that do not achieve remission, even with rigorous medical therapy and steroid-resistant cases are defined as refractory UC [1]. Cytokine profiling has recently been used to understand the pathogenesis of this disease [2]. Although the identification of a patient's cytokine profile is useful not only for understanding the pathophysiology but also for selecting an appropriate drug from a number of biological agents, further research is needed [3]. Prostaglandin E-major urinary metabolite (PGE-MUM) is a urinary metabolite of the inflammatory mediator prostaglandin E2 (PGE2) and partially reflects cytokine profiles. However, measurement of PGE-MUM levels is difficult in clinical settings.

High PGE-MUM levels are observed in patients with active UC [4], suggesting their potential as novel disease biomarkers. Although PGE-MUM levels may be advantageous for improving UC treatment, there are only a few reports on its clinical application. Here, we report our experience with a PGE-MUM measurement-guided treatment.

## Case presentation

**Figure 1:**
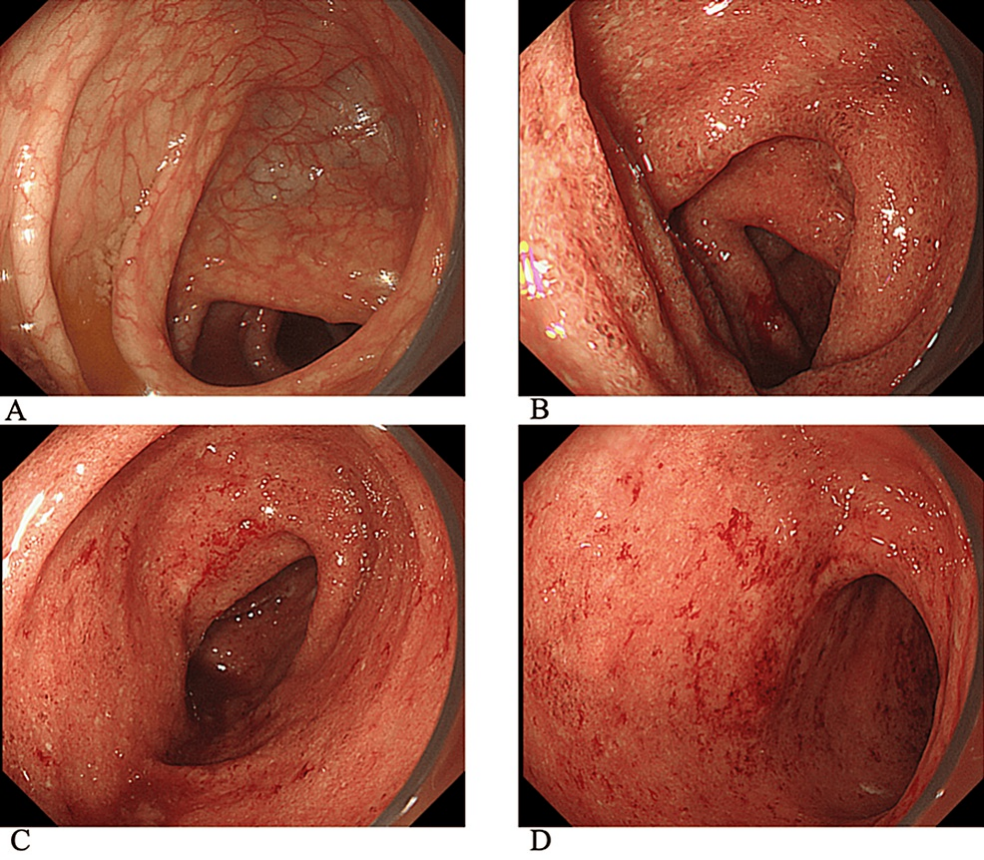
Findings of colonoscopy performed at a previous hospital. (A) Ascending colon, (B) transverse colon, (C) descending colon, and (D) sigmoid colon. No inflammation was observed in the ascending colon, but inflammatory findings, such as granular mucosa and erosions, are observed continuously from the transverse colon to the rectum.

A 14-year-old boy presented to a local hospital with bloody stools and abdominal pain that had persisted for approximately three months. He had no apparent medical history, and no family members had a history of gastrointestinal diseases. Colonoscopy, which was observed at the end of the ileum, revealed continuous mucosal inflammation extending from the rectum to the transverse colon (Figures 1A-1D).

He was diagnosed with UC after a negative stool culture, negative rapid viral antigen test, negative clostridial toxin, and negative cytomegalovirus antibody results, infection was ruled out. He received standard treatment with 5-ASA, Budesonide foam, and 60 mg prednisolone. However, his symptoms worsened, and he was transferred to our hospital.

On admission, body temperature was 37.0 °C, height was 163.0 cm, and weight was 44 kg (48 kg before the illness). The abdomen was flat, soft, and tender, and bowel sounds were diminished. The anus showed no obvious skin tags, hemorrhoidal fistulas, or abscess scarring. No skin manifestations were observed. The blood test results were as follows: white blood cell count was elevated to 15,000/μL, erythrocyte sedimentation rate was 16 mm/h, C-reactive protein was 0.17 mg/dL, and inflammatory markers were hardly elevated. Albumin and hemoglobin levels were 3.7 and 10.3 g/dL, respectively, indicating mild anemia and hypoproteinemia. The patient was diagnosed with pan-colitis-type UC based on colonoscopy findings from the previous physician. Endoscopic severity was 2 (moderate) according to the Mayo Endoscopic Score (MES) [5].

He had severe abdominal pain and seven to eight bloody stools per day, and the Pediatric Ulcerative Colitis Activity Index (PUCAI) [6] score was 75 (severe). As prednisolone was ineffective, early initiation of a biological agent and reduction of the prednisolone dose were attempted. Infliximab and azathioprine were administered after excluding infectious diseases. The patient experienced slight clinical improvement, and his PUCAI score decreased to 60. However, he developed severe abdominal pain and frequent bloody stools. The stool frequency increased to 30 times/day, and his daily activities were limited owing to severe abdominal pain. The patient’s general condition progressively deteriorated.

A second dose of infliximab was administered when his PUCAI score was 80 (the most severe), but it had no effect, and his symptoms did not improve. Although the surgeon strongly recommended total colectomy with a stoma, he refused surgical treatment. Granulocytapheresis was an option to improve the inflammation, but it was challenging because of his poor general condition. He was started on tacrolimus but had difficulty increasing blood tacrolimus concentration due to severe diarrhea. As his blood tacrolimus concentration gradually increased, his clinical symptoms improved and his PUCAI score decreased to 50.

To select the second biological agent, we measured PGE-MUM levels. The PGE-MUM value was 264 μg/gCr (normal value: ≤ 30.2 μg/gCr), which was notably elevated compared with that in a previous study in children [7]. This suggests that his cytokine profile would be of the Th17 cell-dominant type and that drugs inhibiting IL-23 would be appropriate. Therefore, mirikizumab, an antibody against IL-23, was administered as the secondary biological agent. After administration, the desired effect was achieved in a relatively short period of time, with the PUCAI score dropping from 20 to 10, and then to 0. The patient achieved clinical remission and was discharged from the hospital. The patient continued to receive mirikizumab. Tacrolimus was discontinued three months after the initiation of mirikizumab. The patient achieved long-term remission with mirikizumab, 5-ASA, and azathioprine.

**Figure 2:**
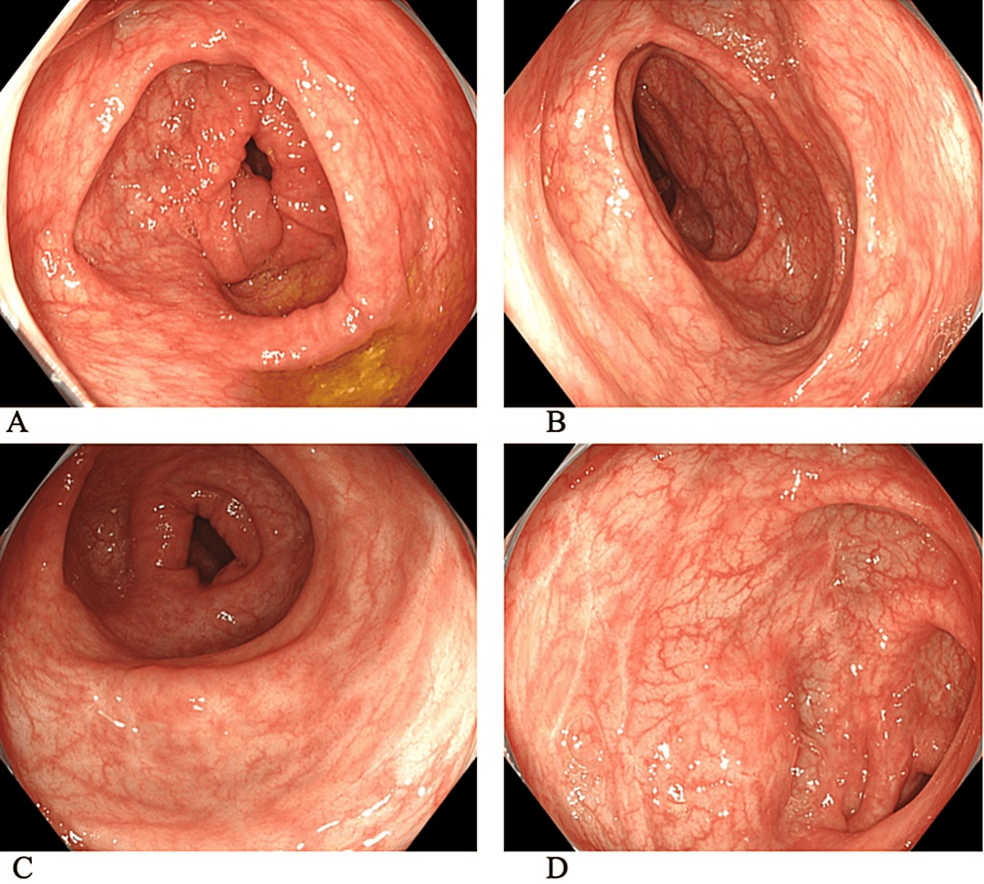
Colonoscopy findings after treatment performed at our hospital. Colonoscopy was performed at our hospital during the remission phase of mirikizumab. (A) Ascending colon, (B) transverse colon, (C) is the descending colon, and (D) sigmoid colon. There is no evidence of mucosal inflammation in any part of the colon. However, linear mucosal atrophy was observed in the descending colon and sigmoid colon, which appeared to be an ulcer scar. These findings suggest the intensity of inflammation during the acute phase.

A colonoscopy was performed two months after discharge. No mucosal inflammation was observed at the end of the ileum or throughout the colon, indicating endoscopic remission with an MES of 0 (Figures 2A-2D).

Pathological examination revealed almost no inflammatory cell infiltration and histological remission was also confirmed. Five months after discharge, the patient’s PGE-MUM levels had decreased to 16.0 μg/gCr.

## Discussion

UC is a chronic inflammatory disease affecting a growing patient population, including children and young adults. UC patients have diverse cytokine profiles [2]. Their profiles fall into three major categories, Th1, Th2, and Th17, based on the major cytokines in each category [3]. These profiles vary from individual to individual, may change with disease stage, and are thought to overlap. Identification of cytokine profiles in patients will be useful for selecting specific therapeutic regimens suitable for each patient. However, the measurement of cytokine profiles is not easily performed in clinical settings.

PGE2 acts on dendritic cells to stimulate the production of IL-23, which, in turn, promotes the proliferation of Th17 cells [8]. PGE2 is rapidly metabolized in the blood, making it challenging to accurately measure its levels. In contrast, PGE-MUM, a metabolite of PGE2, is relatively stable and readily quantifiable. Thus, the measurement of PGE-MUM is an attractive method for cytokine profiling. Theoretically, when PGE-MUM levels are elevated, Th17 levels are considered predominant, and drugs that inhibit IL-23 should be chosen. However, few studies have shown that PGE-MUM levels are useful in the selection of biological agents. In the present case, measurement of PGE-MUM levels led to the determination of the optimal biological agent. We believe that if PGE-MUM levels were not measured, it is quite possible that an appropriate choice of treatment could not have been made.

Our patient had high PGE-MUM levels, suggesting increased PGE2 production. This indicated that our patient had a predominant Th17 pattern of cytokine profile. Therefore, mirikizumab was selected as it targets IL-23, a major cytokine of the Th17 system. Mirikizumab has been shown to improve clinical symptoms even in biologically refractory patients at the 12-week induction and 40-week maintenance phases [9] and to improve patient's quality of life, notably by reducing bowel urgency [10]. Although its administration in children is limited, the use of mirikizumab is likely to increase in the future, along with an accumulation of patients showing its efficacy. At present, the appropriate selection of biological agents for patients with UC is not easy, as various drugs are currently available. In such situations, as in the present case, measurement of PGE-MUM levels contributed to the selection of an appropriate drug for our patient. Although there have been a few similar reports to date, elucidating the usefulness of cytokine profiling in drug selection will be the subject of future studies.

Another candidate is ustekinumab, an IL-12/23p40 monoclonal antibody. Although it could be a treatment option, there is growing evidence to support the preferential use of anti-IL-23 agents in inflammatory bowel disease. Mirikizumab is a better option because of a report showing that selective IL-12 blockade was not beneficial in inflammatory bowel disease [11]. In addition, a study on patients with psoriasis suggested that the inhibition of IL-23 alone was more effective than the combined inhibition of IL-12 and IL-23 [12]. These results indicated that mirikizumab was an appropriate choice for our patient.

## Conclusions

UC that does not improve with appropriate medical therapy is defined as refractory and its treatment is sometimes difficult. We treated a 14-year-old boy with refractory UC. He was considered difficult to treat due to poor response to several medications. His symptoms gradually improved with tacrolimus, followed by successful remission induction and maintenance treatment with mirikizumab. Due to its efficacy, surgical treatment was avoided. In this case, PGE-MUM values were useful in determining the appropriate biological agent.
